# Cross-well machine learning prediction of sonic logs in Newfoundland and Labrador

**DOI:** 10.1038/s41598-026-36053-9

**Published:** 2026-01-15

**Authors:** Bahare Zare, Mohammad Mojammel Huque, Lesley A. James, Hamid Usefi

**Affiliations:** 1 Department of Computer Science, Memorial University of Newfoundland, St. John’s, NL Canada; 2 Department of Process Engineering, Memorial University of Newfoundland, St. John’s, NL Canada; 3 Department of Mathematics and Statistics, Memorial University of Newfoundland, St. John’s, NL Canada

**Keywords:** Energy science and technology, Engineering, Mathematics and computing, Solid Earth sciences

## Abstract

Predicting compressional slowness (DTCO) from non-sonic logs can reduce acquisition cost, fill data gaps, and support field planning. We evaluate blind cross-well DTCO prediction on two offshore Newfoundland & Labrador wells using a strictly leakage-free, features-only strategy: causal lag windows are built from past non-sonic logs and all sonic/sonic-derived channels are excluded. The pipeline includes deterministic depth conditioning, relative-depth features, multi-scale depth derivatives, rank-aggregated feature selection, and time-aware validation on the training well. We compare three model families: Random Forest (RF), Extreme Gradient Boosting (XGBoost), and a BiLSTM. In this setting, tuned XGBoost with the top 20 predictors and a 10-sample lag attains blind cross-well performance of $R^2=0.895$, MAE$=11.38~\mu \mathrm {s/m}$, RMSE$=15.12~\mu \mathrm {s/m}$ when trained on Well 1 and tested on Well 2; the reverse direction is lower, indicating inter-well distribution shift. RF performs competitively in several configurations, whereas BiLSTM underperforms on these data. Overall, rigorous leakage control, depth-aware feature engineering, and principled feature selection are key drivers of performance, and tree-based ensembles provide strong, data-efficient baselines for cross-well pseudo-sonic prediction.

## Introduction

Sonic (acoustic) logs measure elastic–wave travel time and yield high-resolution velocity profiles essential for reservoir characterization, well planning, and geomechanics^1–4^. In practice, sonic tools are not always available due to cost, time, or adverse hole conditions, motivating prediction of compressional slowness (DTCO) from routinely acquired non–sonic logs (e.g., gamma ray, resistivity, drilling parameters) to produce pseudo–sonic curves^4,5^.

Early approaches relied on empirical rock-physics relations and linear regression (e.g., Wyllie’s time-average equation; multi-attribute regression)^6,7^. These methods are simple and interpretable but require extensive re-calibration and degrade in heterogeneous settings^8,9^. Recent studies show improved accuracy with machine learning (ML), including tree ensembles (Random Forest, gradient boosting) and sequence models (LSTM), which capture nonlinear and depth-local relationships in tabular well logs^10–14^. Integrating domain features or auxiliary data (e.g., seismic attributes) can further enhance performance^15^. Parallel work in petrophysical property modeling similarly finds that ensemble methods and carefully tuned ML pipelines are robust for subsurface tabular data^16–21^.

DTCO workflows have also benefited from recent generative and foundation-model advances. Ensemble GANs have improved anomaly detection in compressional sonic logs^24^, and sequence-based GANs have been proposed for synthetic log generation and imputation to support gap filling and data augmentation^25^. In parallel, time-series foundation models (Transformer-based) have been explored for well-log forecasting and anomaly detection^26^, though validation remains basin-specific. These directions point to practical opportunities for quality control, missing-log imputation, and cross-well transfer, while highlighting the importance of rigorous out-of-basin evaluation.

An exciting frontier in formation evaluation is the real-time prediction of sonic logs during drilling operations. Conventionally, sonic logs are recorded via wireline runs after drilling or through logging-while-drilling (LWD) tools that provide data shortly after drilling^28^. However, scenarios exist where neither is available due to cost or hole conditions, yet real-time sonic information would be invaluable for seismic calibration, geosteering, or borehole stability assessment^29^. Machine learning enables this capability by leveraging streaming drilling data to infer sonic properties^30^. The challenge is substantial: real-time models must work with limited measurements (rate of penetration, weight on bit, torque, mud properties) that are noisy and indirectly related to formation acoustic properties^31,32^.

Despite these advances, three methodological gaps remain common: *(i)* incomplete leakage control (e.g., scaling or feature selection using information from the test well, or inclusion of sonic-derived inputs), *(ii)* limited blind cross–well evaluation to quantify generalization under inter–well distribution shift, and *(iii)* insufficient ablations to identify which preprocessing choices (outlier handling, feature generation/selection, temporal context) actually drive performance. Additional issues include the use of random (non–causal) splits on depth-indexed data and reporting metrics in normalized units rather than physical units, both of which can overstate practical accuracy^22,23,27^.

We address these gaps by evaluating leakage-free, blind cross–well DTCO prediction on two offshore Newfoundland & Labrador wells using *features-only, causal lag windows*: inputs contain past non–sonic logs only (no target values) to preclude target leakage by construction. The pipeline applies deterministic depth conditioning, relative-depth features, multi-scale derivatives, and rank-aggregated feature selection; all normalizers and selection are fit on the *training well only* and transferred unchanged to the blind test well. We compare Random Forest (RF), XGBoost (XGB), and BiLSTM under a time-aware validation protocol and conduct ablations over outlier removal, feature generation/selection, and temporal windowing. Results show that disciplined preprocessing and feature selection are the dominant contributors to cross–well accuracy, and that tree ensembles provide strong, data-efficient baselines that outperform LSTM variants under well-to-well shift while training substantially faster.

**Contributions.** (1) A leakage-free pipeline for pseudo–sonic prediction using features-only causal windows; (2) depth-aware feature engineering and robust rank-aggregated selection fit on the training well only; (3) rigorous blind cross–well evaluation in both directions with ablations isolating the impact of outlier handling, feature generation/selection, and temporal context.

## Material and method

### Workflow overview

**Fig. 1:**
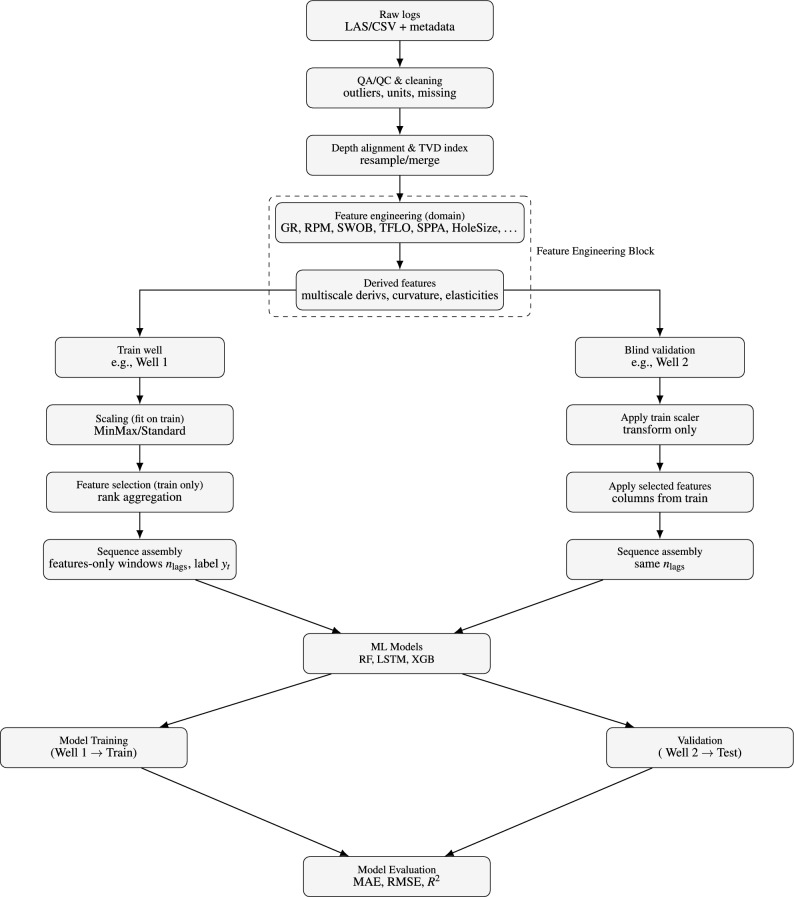
Workflow for blind time series prediction from well log data: preprocessing, sequence generation, model training (RF, LSTM, XGBoost), blind validation, and evaluation.

This subsection outlines the end-to-end pipeline used in all experiments. Figure 1 provides a high-level overview; the remainder of this section describes each stage in turn.

### Data availability and preprocessing

The dataset used in this study includes well log measurements from two wells drilled offshore Newfoundland and Labrador, Canada. Initially, Well 1 contained 55,575 samples with 42 features, and Well 2 had 68,273 samples with 49 features. Only columns common to both datasets were retained, resulting in a total of 31 features. The Geological Formation column was excluded due to having approximately 65% missing values in Well 1.

The target variable, DTCO_MH_R (hereafter referred to as DTCO), is a high-resolution compressional slowness ($\Delta T$) log derived from sonic measurements. To ensure data integrity, any observations with missing (NaN) target values were excluded instead of imputed, guaranteeing that all target measurements correspond to actual observed data.

We predict compressional slowness (DTCO) at depth index *t* using only non–sonic information and a strictly exogenous feature set. To preclude target leakage and reduce redundancy, we remove all sonic or sonic–derived quantities–explicitly including DTCO_INV, DTSH_QPINV, CHSH_QPINV, TISH_QPINV_R6, FRQMAX_QPINV, FRQMIN_QPINV, PR, VPVS, TICO_MH_R6–as well as UCS (often computed from sonic) and any related acoustic impedance or dynamic elastic moduli. We also exclude DEVI (well deviation) because it encodes borehole trajectory rather than formation response and is highly collinear with depth; we retain TVD as the canonical depth coordinate. Tool–specific QC/engineering setpoints are similarly dropped because they reflect operational control rather than rock properties. The retained inputs comprise depth/survey (TVD); drilling and petrophysical logs (GR, ROP5, RPM, SPPA, STOR, SWOB, TFLO, SMSE); and geometry/categorical parameters (HoleSize, A40H, P16H, P28H, P40H). All exclusions were decided *a priori* from data dictionaries and standard petrophysical relationships, and only non–sonic logs (plus their causal, depth–aware derivatives) are used for modeling.

Descriptive statistics for the non–sonic feature set are reported in Supplementary Tables S1–S2. The wells differ markedly in operating regime and depth coverage: Well 2 is shallower on average (TVD: $1645.5\pm 182.4$ m vs. $1811.0\pm 441.6$ m) and exhibits substantially higher surface pressure and flow (SPPA: $28.5\pm 4.3$ kpsi vs. $14.3\pm 1.2$ kpsi; TFLO: $4130.6\pm 288.6$ vs. $2742.9\pm 870.8$), while Well 1 shows broader dispersion in several logs (e.g., GR: $91.1\pm 34.4$ vs. $84.3\pm 16.7$; ROP5: $28.3\pm 14.6$ vs. $44.9\pm 11.0$). Deviation and torque/pressure proxies also shift (DEVI: $80.1\pm 4.8$ in Well 2 vs. $42.8\pm 13.5$ in Well 1; SMSE is orders of magnitude larger and more variable in Well 2). Despite broadly similar DTCO medians (Well 1: 316.7 $\mu$s/m; Well 2: 312.3 $\mu$s/m), the spread differs (std: 60.8 vs. 47.3 $\mu$s/m), indicating a nontrivial distribution shift. These contrasts motivate our blind cross–well protocol, train–well–only scaling, and explicit ablations to assess generalization across wells.

### Outliers removal

To suppress spurious sensor excursions while preserving temporal (depth) alignment, we apply a leakage–free interquartile masking on *sensor* channels only. Let $\mathcal {S}$ denote the set of non–sonic sensor logs (e.g., GR, SPPA, RPM,...), *z* denote true vertical depth (TVD), and *y* the target (DTCO_MH_R). We first sort the training well by *z* and compute, for each sensor $s\in \mathcal {S}$, the quartiles $Q_1(s)$ and $Q_3(s)$ and the interquartile range $\textrm{IQR}(s)=Q_3(s)-Q_1(s)$. Fixed bounds

$$\textrm{LOW}(s)=Q_1(s)-1.5\,\textrm{IQR}(s),\qquad \textrm{HIGH}(s)=Q_3(s)+1.5\,\textrm{IQR}(s)$$

are estimated *only on the training well* and then applied unchanged to both the training and test wells. Any value of sensor *s* outside $[\textrm{LOW}(s),\textrm{HIGH}(s)]$ is masked to NaN. We do *not* IQR–filter TVD or the target; both remain untouched and rows are *not* dropped, thereby keeping all indices aligned for lagged window construction. Masked sensor values are imputed causally along depth using forward–fill. Finally, TVD serves as the depth index for plotting and alignment; depth–aware features (e.g., relative depth, causal multi–scale derivatives) are derived from the cleaned sensors as explained in the next section. This procedure prevents target/test leakage because the thresholds are fit on the training well only, while preserving the original sampling grid for sequence modeling.

### Feature engineering

Both wells were processed with an identical, deterministic pipeline. First, records were coerced to numeric TVD, sorted, and indexed by TVD to enforce a strictly monotonic depth axis. Second, relative depth within each HoleSize segment ($z_{\textrm{rel}}\in [0,1]$) was computed to capture intra-segment trends and reduce discontinuities at bit-size changes. Third, depth-aware features were generated causally (using only past and current samples) for each retained predictor: multi-scale Savitzky–Golay smoothing (window lengths 9, 21, 41; polynomial order 2), first and second derivatives with respect to TVD (slope and curvature), log-gradients (elasticities) with small positive clipping for numerical stability, polynomial bases of $z_{\textrm{rel}}$ and their depth-gradients, and exponentially weighted means (EWM; $\alpha =0.25$). This procedure yields a depth-consistent, leakage-free design matrix that preserves local structure at multiple scales while remaining comparable across wells.

### Feature selection with SVM, RF, and XGBoost

Feature selection was performed exclusively on the training well to ensure external validity. After a light prefilter that removed quasi-constant variables and pruned near-duplicate features by retaining, within any highly correlated pair ($|\rho |\ge 0.98$), the variable with larger mutual information with DTCO, three complementary rankings were produced: random-forest permutation-free importance, XGBoost split-gain importance, and a standardized LinearSVR embedded in recursive feature elimination (RFE). Rankings were aggregated by averaging normalized ranks (Borda method) to obtain a single ordered list of predictors. The number of selected features *K* was chosen on a time-aware validation split (last 20% of the training well) by training an XGBoost model on the top-*K* features and selecting the *K* that minimized validation RMSE. The final model was refit on the full training well using the chosen subset and then evaluated on the external well. All smoothing and statistics were computed causally to prevent look-ahead.

**Algorithm 1:**
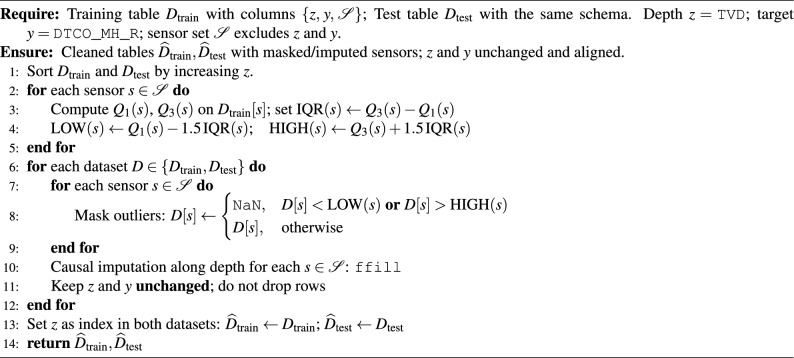
Outlier handling without target or depth leakage (sensors only).

###  Normalization

Because the input variables span different ranges (Tables S1–S2), we applied *min–max* scaling fitted only on the training well and then reused those fitted transformers to scale both the training and external test wells. Concretely, we trained two independent MinMaxScaler objects on Train dataset: one for the feature matrix $\textbf{X}$ and one for the target *y*. The same fitted scalers were then applied to Validation dataset. For any feature *x*, scaling was

$$x_{\text {scaled}} \;=\; \frac{x - x_{\min ,\textrm{train}}}{x_{\max ,\textrm{train}} - x_{\min ,\textrm{train}}}\,,$$

with $x_{\min ,\textrm{train}}$ and $x_{\max ,\textrm{train}}$ computed on Train dataset only; the target *y* was scaled analogously using its own scaler.

### Sequence construction (features–only, causal)

We adopt a strictly exogenous, leakage–free sequence construction based on *features only*. For a chosen lag length *L*, the input at depth index *t* is the feature window

$$\textbf{X}_{t-L+1:t}=\big (\textbf{x}_{t-L+1},\ldots ,\textbf{x}_{t}\big )\in \mathbb {R}^{L\times d},$$

and the supervised target is the contemporaneous label $y_t$. The mapping

$$y_t \;=\; g\!\big (\textbf{X}_{t-L+1:t}\big )$$

uses no past (or future) values of the target in the inputs, thereby eliminating any path for target leakage and preserving depth–causality. Windows are formed on the training well only; all scalers are fit on the training well and applied unchanged to the validation well. The lag length *L* is selected via time–aware validation. Pseudocode for this construction is provided in Algorithm 2.

**Algorithm 2:**
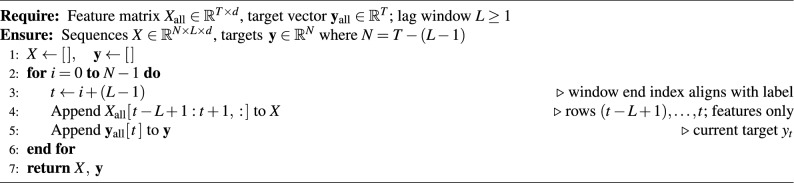
Features-Only Lag Windows (no target in inputs).

## Machine learning algorithms

Our modeling strategy is based on blind predictions: the model is trained on data from one well and validated on data from a different well to rigorously evaluate generalization performance. Specifically, we train on Well 1 and report predictions on Well 2, and vice versa. By following this protocol and ensuring feature scaling is conducted independently for each well, we eliminate information leakage and obtain a realistic measure of predictive accuracy for unseen geological intervals.

We employ three machine learning algorithms in our experiments: Random Forest (RF)^33^, Long Short-Term Memory (LSTM) neural networks^34^, and eXtreme Gradient Boosting (XGBoost)^35^. RF serves as a robust ensemble method based on decision trees, LSTM networks are well-suited for capturing temporal dependencies in sequential data, and XGBoost is a high-performance gradient boosting algorithm known for its predictive accuracy. By comparing these diverse models, we aim to rigorously evaluate performance and highlight the strengths of each approach for time series prediction in well log analysis.

Random Forest fits trees independently and parallelizes well across CPU cores; its wall–clock time scales roughly with the number of trees $T$, the number of training samples $N$ (after windowing/lagging and masking), and the effective feature dimensionality $d_{\textrm{eff}}$ actually considered at each split (post feature engineering/selection), yielding $\mathcal {O}\!\left( T\,N\log N\,d_{\textrm{eff}}\right)$ for approximately balanced trees. In contrast, XGB builds trees sequentially via boosting (parallelizing within each tree); with the histogram builder and early stopping, the practical cost is $\mathcal {O}\!\left( B\,N\log N\,d_{\textrm{eff}}\right)$, where $B$ is the (stopped) number of boosting rounds. In our pipeline, RF typically converges with a few hundred trees and is faster on multi-core CPUs, whereas XGB uses fewer, deeper trees and often achieves higher accuracy per tree at modest additional time. Prediction latency for both models is low, and memory usage grows with the total node count (trees $\times$ depth).

Subsequently, several evaluation metrics were computed on the validation data to rigorously assess model performance. The MAE measures the average absolute difference between predicted and actual values, providing an interpretable indication of typical error magnitude. The coefficient of determination $R^2$ quantifies the proportion of variance in the target variable that is explained by the model, with values closer to 1 indicating better fit. The mean squared error (MSE) computes the average squared difference between predicted and true values, placing greater emphasis on larger errors. Finally, RMSE is the square root of MSE, retaining the original units of the target variable and penalizing larger errors more strongly. Each term is calculated as follows.

$$\begin{aligned}&MAE = \frac{1}{n} \sum _{i=1}^{n} |y_i - \hat{y}_i|, \quad MSE = \frac{1}{n} \sum _{i=1}^{n} (y_i - \hat{y}_i)^2, \\&RMSE = \sqrt{MSE}, \quad R^2 = 1 - \frac{\sum _{i=1}^{n} (y_i - \hat{y}_i)^2}{\sum _{i=1}^{n} (y_i - \bar{y})^2} \end{aligned}$$

where $y_i$ is the observed value, $\hat{y}_i$ is the predicted value, $\bar{y}$ is the mean of observed values, and *n* is the number of samples. All metrics were evaluated exclusively on the validation set to ensure a fair assessment of the model’s generalization ability. For interpretability, predictions and ground truth values were transformed back to their original scale using the fitted target scaler.

The machine learning models were implemented using Python (version 3.11) with TensorFlow library (version 2.7.0) on the Jupyter Notebook platform.

### Random forest (RF)

RF is an ensemble learning technique that builds multiple decision trees during training and combines their outputs for improved accuracy and robustness. It works by training each tree on a random subset of the training data and then combining their predictions during the testing phase. Known for its versatility and effectiveness in handling complex datasets, RF is widely used for classification and regression tasks in machine learning^36–38^.

First, the scaled training and validation datasets were converted into supervised learning format by generating lagged sequences of length $n_{\text {lags}} = 13$, such that each input sample consists of feature vectors from the preceding thirteen time steps. Since RF models require two-dimensional input, the resulting three-dimensional arrays were flattened along the lagged time steps, yielding input matrices of size $[n_{\text {samples}}, n_{\text {lags}} \times n_{\text {features}}]$ for both the training and validation sets.

The RF model was trained using 100 estimators and a fixed random seed of 42 to ensure reproducibility. After training, predictions were made on the validation set, and the predicted values were inverse-transformed using the previously fitted scaler to restore the original scale of the target variable. Model performance was then evaluated on the validation set using the evaluation metrics mentioned above.

### eXtreme gradient boosting

The Extreme Gradient Boosting (XGBoost) algorithm, a powerful machine learning method, has proven effective in handling missing data and improving weak models to create more accurate predictions. As a refined version of the Gradient Boosting Decision Tree, XGBoost has shown impressive results in various applications due to its accuracy, speed, and efficient data processing.

We experimented with various combinations of hyperparameters in our XGBoost regressor, based on the *GridSearchCV* from the *scikitlearn* library; the most effective hyperparameters were the number of estimators and learning rate. Specifically, we tested with 1000, 2000, 5000, and 10000 estimators, along with learning rates of 0.01, 0.05, 0.1, and 0.3. Ultimately, we initialized the XGBoost regressor with a specific configuration: 1000 estimators, learning rate of 0.05, and the maximum depth (limited to 20).

### Long short-term memory

To model the sequential dependencies in the well log data, we implemented a deep bidirectional long short-term memory (LSTM) neural network. The model architecture comprises an initial bidirectional LSTM layer with 200 units and Glorot uniform initialization, followed by a dense layer with 20 units and a tanh activation function. This is succeeded by a second bidirectional LSTM layer with 150 units, and two additional dense layers (each with 20 units and tanh activation). To reduce overfitting, a dropout layer with a rate of 0.25 is included. The final output layer consists of a single neuron for regression.

We trained the model using the Adam optimizer with a learning rate of 0.001 and gradient clipping (clipnorm=1.0) to enhance training stability. The mean squared error (MSE) loss function was used to optimize the network parameters. Model training was performed for 70 epochs with a batch size of 72, using the training dataset from one well and validating on the other (blind prediction). Early stopping based on validation loss can be optionally employed to prevent overfitting.

After training, we evaluated model performance on the validation set by computing standard regression metrics mentioned above.

## Results

This section evaluates the proposed framework on real well–log data under a blind cross–well protocol: models are trained on one well and evaluated on the other. To prevent leakage, *all* feature selection, scaling, and hyperparameter tuning are performed *exclusively* on the training well; the opposite well is used only for held–out testing. We report metrics and TVD–aligned visualizations on these held–out sets, organizing results to show (i) the benefit of feature selection, (ii) the effect of sequence (lagged) representations, and (iii) cross–well generalization across distinct geological intervals.

For the Random Forest (RF) model, we used a time–aware validation split within the training well (first 80% for fitting, final 20% for validation), then refit on the full training well with the selected configuration before testing on the other well. The search jointly optimized the number of retained features (*K*), the lag window ($n_{\text {lags}}$), and RF hyperparameters. The resulting optimum are reported in Table 1. We observed only minor variations in the tuned settings when swapping the training well (Well 1 vs. Well 2), indicating a stable optimum under the blind validation protocol.

**Table 1:** Final hyperparameters and data settings used in blind cross–well runs (time–aware tuning on the training well). “–” denotes not applicable.

**Setting**	**RF**	**XGB**
TOP_K (features)	30	20
$n_{\text {lags}}$ (samples)	10	10
$n_{\text {estimators}}$/rounds	600	$\sim$243 (early stop)
max_depth	None	4
min_samples_leaf/min_child_weight	2	8
max_features/colsample_bytree	sqrt	0.8
bootstrap	False	–
subsample	–	0.9
learning rate $\eta$	–	0.03
$\lambda$ ($\ell _2$ reg.)	–	1.0
tree_method	–	hist
eval_metric (early stop)	–	RMSE
random_state/seed	42	42

Table 2 presents the performance of the RF model under a cross-well validation protocol, where training and testing were alternately performed on Well 1 and Well 2.

**Table 2:** Random Forest cross-well blind validation results: models are trained on one well (Well 1 or Well 2) and evaluated on the other.

**Train** $\rightarrow$ **Validation**	**MAE**	**RMSE**	$\mathbf {R^2}$
Well 1 $\rightarrow$ Well 2	21.10	25.43	0.71
Well 2 $\rightarrow$ Well 1	25.10	30.84	0.72

**Fig. 2:**
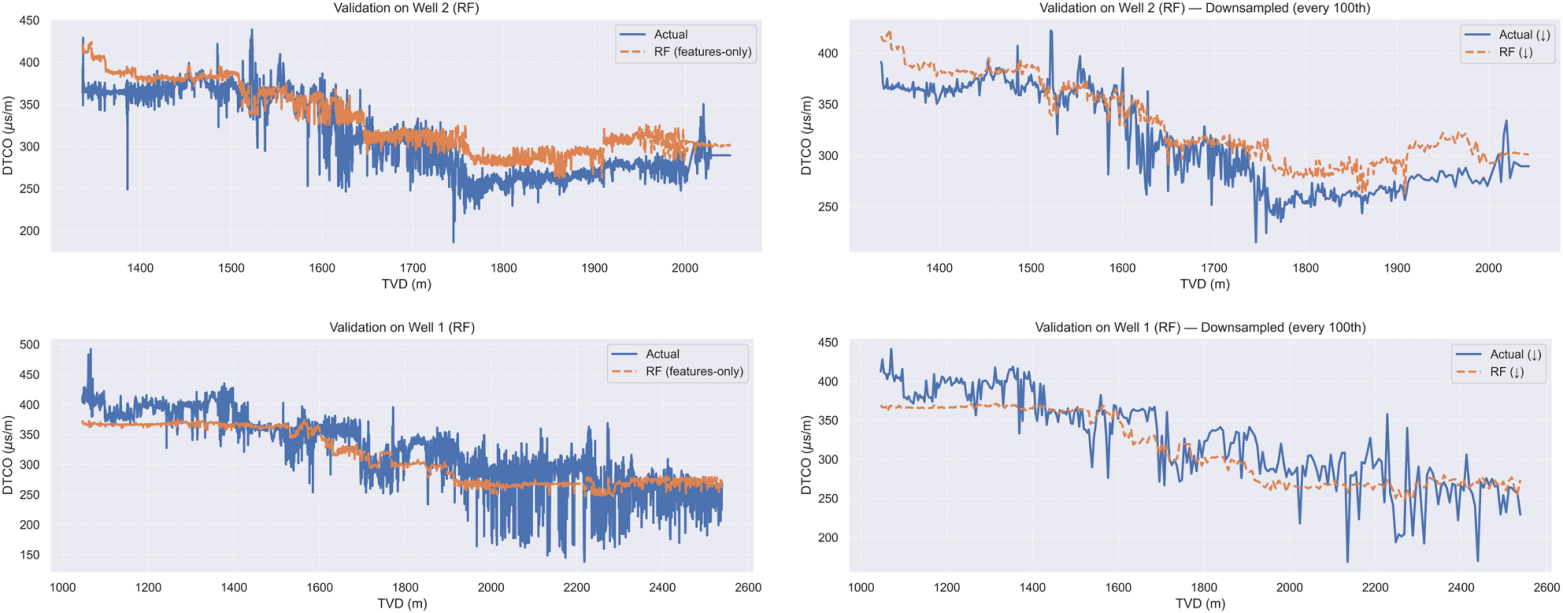
Random Forest model results for blind cross-well validation. Top row: Training on Well 1 and validation on Well 2; bottom row: Training on Well 2 and validation on Well 1. (Left:) Full validation set, showing predicted versus actual DTCO. (Right:) Same comparison, but plotted for every 100th data point to improve visual clarity.

Figure 2 illustrates the performance of the RF model under the blind cross-well validation protocol. The top row displays results when the model is trained on Well 1 and validated on Well 2, while the bottom row shows the reverse. In each case, predicted and actual DTCO values are plotted across the entire validation interval (left panels), as well as for a sparsified subset (right panels) to enhance readability. The close alignment between predicted and observed values across both wells demonstrates the model’s strong predictive accuracy and robustness in cross-well applications.

For XGBoost (XGB), we adopted the same blind cross–well protocol and time–aware split on the training well (first 80% for fitting, final 20% for validation). The search jointly optimized the retained feature count (*K*), the lag window ($n_{\text {lags}}$), and booster hyperparameters with early stopping on validation loss. The selected configuration is reported in Table 1. We then refit the model on the full training well using the chosen features and evaluated it out of sample on the opposite well.

**Fig. 3:**
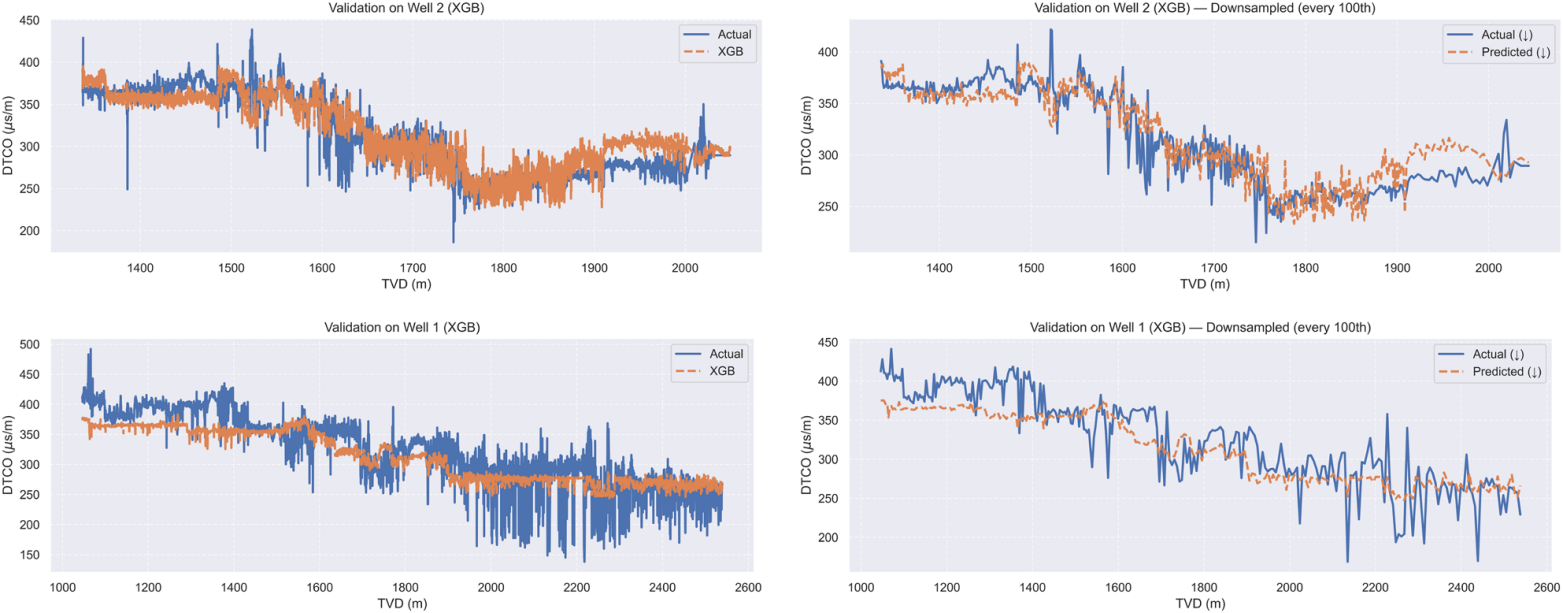
XGB model results for blind cross-well validation. Top row: Training on Well 1 and validation on Well 2; bottom row: Training on Well 2 and validation on Well 1. (Left:) Full validation set, showing predicted versus actual DTCO. (Right:) Same comparison, but plotted for every 100th data point to improve visual clarity.

Table 3 reports blind cross–well performance for XGB. Training on Well 1 and validating on Well 2 yields substantially lower errors than the reverse direction, indicating asymmetric generalization and a distribution shift between the two wells. Figure 3 visualizes the predictions versus true DTCO aligned by TVD foron the validation datasets.

**Table 3:** XGB cross-well blind validation results: models are trained on one well (Well 1 or Well 2) and evaluated on the other.

**Train** $\rightarrow$ **Validation**	**MAE**	**RMSE**	$\mathbf {R^2}$
Well 1 $\rightarrow$ Well 2	14.71	18.75	0.84
Well 2 $\rightarrow$ Well 1	25.94	32.23	0.70

Tables 2 and 3 report blind cross–well results for RF and XGB. XGB achieves lower errors and higher $R^2$ overall, while RF is slightly more stable in noisy intervals. The consistent asymmetry between Well 1$\rightarrow$Well 2 and Well 2$\rightarrow$Well 1 across both models indicates distribution shift rather than a model–specific artifact, likely driven by (i) covariate shift in non–sonic predictors (different depth ranges and operating regimes), (ii) facies mix differences altering the DTCO–feature relationship, and (iii) sensor/QC discrepancies affecting signal–to–noise. Both methods were tuned over retained features and lag length; XGB additionally benefits from booster hyperparameters (learning rate, depth, subsampling), yielding the top scores. Practical mitigations include formation-aware tuning, stability checks of feature importance across wells, simple covariate-shift diagnostics, and lightweight domain-adaptation or direction-specific ensembling.

Table 4 reports blind cross–well results for standard regression baselines trained on Well 1 and evaluated on Well 2. We followed the same set up by using our features-only lag windows and fitting the scaling transformation from the train dataset (Well 1). Regularized linear models perform best under the distribution shift: ElasticNetCV and LassoCV achieve the highest test $R^2$ (0.651 and 0.646, respectively) with MAE $\approx$ 21–22 $\mu$s/m and RMSE $\approx$ 28 $\mu$s/m, followed by RidgeCV ($R^2=0.592$). Plain linear regression is markedly weaker ($R^2=0.374$), underscoring the value of shrinkage. Nonparametric methods are less robust: SVR (RBF) degrades to $R^2=0.287$, and KNN collapses to negative $R^2$ despite near-perfect resubstitution on the train well, illustrating severe overfitting to local depth neighborhoods.

**Table 4:** Baseline models under blind cross–well evaluation (train: Well 1; test: Well 2). All models use features-only lag windows and train-well–only scaling. Metrics reported on the held-out Test well; errors in $\mu$s/m.

**Model**	**MAE**	**RMSE**	$\mathbf {R^2}$
ElasticNetCV	21.30	27.97	0.65
LassoCV	21.47	28.16	0.64
RidgeCV	25.25	30.21	0.59
Linear	24.10	37.43	0.37
SVR (RBF)	33.51	39.96	0.28
KNN	38.38	48.59	$-0.05$
LSTM	27.25	34.21	0.54

We explored multiple recurrent architectures, including (i) single- and two–layer LSTMs (32–256 units), (ii) bidirectional LSTMs, and (iii) hybrid Conv1D $\rightarrow$ LSTM front–ends with causal/dilated convolutions. For each, we evaluated different lag windows ($n_{\text {lags}}\in \{13,25,35,50\}$), batch sizes (32–256), losses (MSE and Huber), optimizers (Adam with learning–rate schedules and gradient clipping), and regularization (dropout, recurrent dropout, and L2 weight decay). We also tested z–score versus min–max normalization (fit on the training well only) and time–aware early stopping using the last 20% of the training well. Despite these ablations, LSTM variants consistently underperformed the tree ensembles under blind cross–well validation, yielding substantially lower $R^2$ and higher MAE/RMSE than RF/XGB. We attribute this principally to the limited number of wells (domain shift across wells), strong nonstationarity with depth, and the relatively modest data regime for training high–capacity sequence models.

## Ablation study

We ablate three preprocessing blocks to measure their effect on blind cross–well prediction: **Outlier removal (OutRm)** suppresses tool artifacts and unit errors, stabilizing distributions for train-only scaling; **Feature generation/selection (FG/FS)** adds depth-aware constructs (relative depth, multi-scale derivatives/elasticities) and prunes redundancy via MI prefiltering and rank-aggregated top-*K* (RF/XGB/SVR-RFE), reducing variance and proxy leakage; **Temporal (lagged) features** provide strictly causal context from non-sonic logs, with $n_{\text {lags}}$ tuned time-aware on the training well. Table 5 reports RF and XGBoost results for all component combinations (check marks indicate enabled steps) using $R^2$, MAE, and RMSE on the blind well.

Under blind cross–well testing (train on Well 1, test on Well 2), the ablation study in Table 5 shows a clear hierarchy of preprocessing effects. Using *feature generation/selection (FG/FS) with outlier removal (OutRm) and no lags* already delivers strong generalization: RF attains $R^2=0.73$ (MAE$=19.06$, RMSE$=24.94$) and XGB reaches $R^2=0.81$ (MAE$=15.24$, RMSE$=20.93$). Compared to the fully tuned cross-well pipelines reported in Table 3 (e.g., XGB $\approx 0.84$ for Well 1$\rightarrow$Well 2), the best ablation variant (FG/FS + OutRm, no lags) approaches but does not match the top score. Overall, the pattern is consistent:

- *(i)* FG/FS is the dominant driver of cross-well generalization;
- *(ii)* temporal features add value only when paired with disciplined preprocessing and can harm otherwise; and
- *(iii)* OutRm is helpful but insufficient in isolation–the gains emerge primarily from careful, leakage-aware variable curation.

The ablation study (Table 5) shows that feature generation/selection (FG/FS) is the dominant driver of cross–well generalization. First, depth–aware generation (relative depth, multi–scale derivatives) raises the signal–to–noise ratio by encoding local trends and removing spurious scale effects. Second, rank–aggregated selection (top–*K*) suppresses redundancy and multicollinearity, which lowers variance and improves split quality for boosted trees, yielding tighter residuals and more stable $R^2$. Third, restricting the model to predictors that are informative in a time–aware split on the *training* well mitigates covariate shift by emphasizing features with portable relationships across wells.

**Table 5:** Ablation study: Cross-well prediction performance of RF and XGB models trained on Well 1 and tested on Well 2 under different preprocessing strategies. Each combination of **outlier removal (OutRm)**, **feature generation and selection (FG/FS)**, and **temporal (lagged) features** is indicated with a check mark. The first two rows report models using only feature selection, the next two use only temporal features, and subsequent rows summarize other combinations.

OutRm	Temporal	FG/FS	Model	R$^2$ Score	MAE	RMSE
✓		✓	RF	0.73	19.06	24.94
✓		✓	XGB	0.81	15.24	20.93
✓	✓		RF	−1.13	63.67	71.57
✓	✓		XGB	−0.04	40.83	47.83
✓			RF	−0.09	38.14	48.64
✓			XGB	−0.60	48.19	59.07
	✓	✓	RF	0.62	24.66	28.74
	✓	✓	XGB	0.71	20.86	25.82
		✓	RF	0.72	20.10	24.32
		✓	XGB	0.69	21.15	26.05
			RF	−0.30	42.95	53.61
			XGB	−0.71	52.12	61.22

## Conclusion

This study systematically assessed the predictive performance of machine learning models for cross-well (blind) sonic log prediction using well log data from two offshore wells in Newfoundland and Labrador, Canada. We evaluated a range of preprocessing strategies, including outlier removal, feature selection, normalization, and temporal (lagged) feature engineering, and analyzed their impact on RF, XGB, and LSTM models.

Our results show that ensemble tree-based methods (RF and XGB) consistently outperformed LSTM networks in both accuracy and computational efficiency. In cross-well validation, RF and XGB achieved higher $R^2$ scores and lower error metrics (MAE, RMSE) than LSTM, while also requiring significantly less training time. This highlights the practical advantages of ensemble methods for sonic log prediction, particularly in settings where computational resources or rapid deployment are important.

We also found that feature generation and selection as well as temporal feature engineering were essential for optimal results. Incorporating lagged (windowed) inputs led to some improvements, underscoring the value of modeling sequential dependencies in well log data. While LSTM models are commonly used for time series forecasting, our experiments indicate that their benefit in this application is limited compared to recent tree-based approaches.

In summary, this work demonstrates the effectiveness of tree-based ensemble models for blind cross-well sonic log prediction and emphasizes the importance of thorough preprocessing and temporal feature construction. The proposed workflow can be used to synthesize DTCO where sonic measurements are missing or unreliable, supporting downstream tasks such as geomechanical modeling (e.g., moduli estimation, pore-pressure/stress profiling), seismic calibration, and well planning without additional wireline runs. Because transforms are fit on a reference well and then applied unchanged to new wells, the procedure enables near–real-time generation of pseudo-sonic logs from routinely acquired drilling/LWD channels, offering a cost- and time-efficient fallback and a practical quality-control layer when measured sonic data are sparse or noisy.

This study is limited by the small number of wells and basin-specific conditions, which constrain statistical power and the scope of generalization. Future work will expand to multi-well, multi-basin evaluations with formation-aware splits, investigate domain adaptation and uncertainty quantification, and assess modern sequence models (e.g., lightweight Transformers) under the same leakage-controlled protocol.

## Supplementary Information

Supplementary Information.

## Data Availability

The datasets used in this paper are available from authors upon reasonable request.

## List of symbols

DTCO: Compressional slowness (target), in $\mu$s/m
TVD: True vertical depth, in m
RF: Random forest
XGB: Extreme gradient boosting (XGBoost)
LSTM/BiLSTM: (Bi-)long short–term memory network
FG/FS: Feature generation/feature selection
OutRm: Outlier removal
EWMA: Exponentially weighted moving average
SG: Savitzky–Golay (trend/derivative filter)
QC: Quality control
CV/LOOCV: Cross-validation/leave-one-out cross-validation
MAE, RMSE, $R^2$: Evaluation metrics (mean absolute error, root mean square error, coefficient of determination)
Well 1/Well 2: Training/validation wells used in blind cross-well tests
GR, SPPA, RPM, STOR, SWOB, TFLO, ROP5, SMSE: Non-sonic log channels used as predictors (standard vendor/channel names as provided)
HoleSize, DEVI, A40H, P16H, P28H, P40H: Geometry/trajectory/tool channels (vendor units); DEVI excluded due to redundancy with depth
