# Supplementary material for: Cross-well machine learning prediction of sonic logs in Newfoundland and Labrador
Source: Sci Rep. 2026 Jan 15;16:5292. doi: 10.1038/s41598-026-36053-9 (PMC12881373; doi:10.1038/s41598-026-36053-9)
Supplement: Supplementary file 1 — Supplementary Information. [file 41598_2026_36053_MOESM1_ESM.pdf]

Supplementary Material

In this section, we provide additional detail supporting the main manuscript.

Table S1. Descriptive statistics of selected features for Well 1

	mean	std	min	25%	50%	75%	max
TVD	1811.0	441.6	1044.9	1416.6	1854.7	2190.9	2539.2
GR	91.1	34.4	8.1	68.3	94.7	111.1	171.0
SPPA	14279.0	1230.2	68.9	13525.0	14082.1	15079.1	17247.3
RPM	156.3	18.0	32.0	149.0	158.7	168.5	181.0
STOR	28.1	12.6	5.3	15.6	26.2	39.6	55.1
SWOB	12.7	5.0	0.0	9.3	12.7	15.6	48.7
TFLO	2742.9	870.8	1662.8	1987.2	2002.9	3713.0	4175.9
ROP5	28.3	14.6	4.7	17.5	24.0	39.5	195.3
SMSE	86498.8	121359.5	6.7	104.9	216.6	193490.8	1900162.0
DEVI	42.8	13.5	29.3	29.9	36.0	60.3	61.0
A40H	3.0	6.5	0.1	2.1	2.5	3.2	200.0
P16H	4.5	38.3	0.1	1.8	2.3	2.9	1000.0
P28H	4.5	36.2	0.1	2.0	2.4	3.0	1000.0
P40H	6.3	47.0	0.1	2.1	2.6	3.1	1000.0
DTCO_MH_R	320.2	60.8	138.2	274.7	316.7	371.3	492.0

Table S2. Descriptive statistics of selected features for Well 2

	mean	std	min	25%	50%	75%	max
TVD	1645.5	182.4	1336.2	1489.2	1642.9	1796.4	2049.5
GR	84.3	16.7	30.1	76.9	86.0	95.4	130.6
SPPA	28488.9	4278.3	12376.6	24632.6	28473.4	31761.8	36755.3
RPM	148.1	13.6	39.0	139.0	150.0	158.5	198.0
STOR	31.9	8.8	7.5	23.3	31.2	40.8	51.2
SWOB	8.5	4.0	0.0	5.4	7.6	11.1	19.6
TFLO	4130.6	288.6	1267.3	4180.2	4189.7	4199.1	4539.6
ROP5	44.9	11.0	0.3	39.6	49.3	54.2	92.9
SMSE	1467040.5	4062612.1	155505.4	858840.8	1102051.0	1623884.8	539697129.6
DEVI	80.1	4.8	56.0	81.2	81.3	81.4	82.1
A40H	5.1	17.9	0.1	2.3	2.9	3.9	200.0
P16H	26.8	326.6	0.1	2.3	3.0	4.1	5000.0
P28H	41.1	418.7	0.2	2.8	3.5	4.7	5000.0
P40H	47.0	448.5	0.4	3.3	4.1	5.4	5000.0
DTCO_MH_R	317.2	47.3	186.3	268.0	312.3	364.6	438.8
